# The Energy Homeostasis Principle: A Naturalistic Approach to Explain the Emergence of Behavior

**DOI:** 10.3389/fnsys.2021.782781

**Published:** 2022-01-06

**Authors:** Sergio Vicencio-Jimenez, Mario Villalobos, Pedro E. Maldonado, Rodrigo C. Vergara

**Affiliations:** ^1^The Center for Hearing and Balance, Otolaryngology-Head and Neck Surgery, Johns Hopkins University School of Medicine, Baltimore, MD, United States; ^2^Escuela de Psicología y Filosofía, Universidad de Tarapacá, Arica, Chile; ^3^Laboratorio de Neurosistemas, Departamento de Neurociencia & BNI, Facultad de Medicina, Universidad de Chile, Santiago, Chile; ^4^Departamento de Kinesiología, Facultad de Artes y Educación Física, Universidad Metropolitana de las Ciencias de la Educación, Ñuñoa, Chile

**Keywords:** homeostasis, free energy principle, behavior, energy, neural network

## Abstract

It is still elusive to explain the emergence of behavior and understanding based on its neural mechanisms. One renowned proposal is the Free Energy Principle (FEP), which uses an information-theoretic framework derived from thermodynamic considerations to describe how behavior and understanding emerge. FEP starts from a whole-organism approach, based on mental states and phenomena, mapping them into the neuronal substrate. An alternative approach, the Energy Homeostasis Principle (EHP), initiates a similar explanatory effort but starts from single-neuron phenomena and builds up to whole-organism behavior and understanding. In this work, we further develop the EHP as a distinct but complementary vision to FEP and try to explain how behavior and understanding would emerge from the local requirements of the neurons. Based on EHP and a strict naturalist approach that sees living beings as physical and deterministic systems, we explain scenarios where learning would emerge without the need for volition or goals. Given these starting points, we state several considerations of how we see the nervous system, particularly the role of the function, purpose, and conception of goal-oriented behavior. We problematize these conceptions, giving an alternative teleology-free framework in which behavior and, ultimately, understanding would still emerge. We reinterpret neural processing by explaining basic learning scenarios up to simple anticipatory behavior. Finally, we end the article with an evolutionary perspective of how this non-goal-oriented behavior appeared. We acknowledge that our proposal, in its current form, is still far from explaining the emergence of understanding. Nonetheless, we set the ground for an alternative neuron-based framework to ultimately explain understanding.

## Introduction

When an animal displays different behaviors, what are the primary processes occurring in the nervous system? How do neurons, neuronal networks, and ultimately the whole nervous system participate in behavior generation? This article argues that the nervous system unfolds autogenous mechanisms of energetic homeostasis, maintaining its energy equilibrium as a system. In our view, the nervous system operates in the continuous present tense of its structural dynamics under strictly local rules of energy stability, without pursuing biological goals or adaptive adjustments for the organism. This spontaneous process of maintaining its energy balance occurs so that under statistically normal anatomical, physiological, and ecological conditions, it results precisely in those behaviors that prove to be adaptive for the animal.

This view of the nervous system corresponds, in essence, to what has been recently introduced as the Energy Homeostasis Principle (EHP; Vergara et al., 2019). This theoretical proposal draws strongly from the autopoietic theory of cognition in the sense of being strictly naturalistic (Maturana, 1978; Villalobos and Ward, 2015; Villalobos, 2015), and resonates, although with important nuances, with some aspects of the Free Energy Principle (FEP) approach in theoretical neuroscience (Friston and Stephan, 2007; Friston, 2010). The EHP does not hold that animal behavior and cognition arise only because the nervous system is a homeostatic energy system. If that were the case, we should observe cognition and complex behavior in any homeostatic energy system, as may occur in an open thermodynamic system that exhibits some degree of stability, such as tornadoes and stars (Ulanowicz and Hannon, 1987; McGregor and Virgo, 2011). Instead, the proposal is to realize that while we observe the behavior or the signs of cognition shown by an organism, its nervous system operates by simply following, in its own way, the EHP.

The nervous system is a homeostatic energy system, like other similar natural systems, but with significant structural and organizational features that make it unique. These features are essential because they explain why the nervous system, despite operating under the EHP, can generate phenomena such as animal behavior and cognition. The argument EHP asserts is that despite all the unique features we may find in the nervous system, it remains the fact that its operations follow, ultimately, homeostatic energy mechanisms.

This latter statement merits further discussion. When we speak of the unique features in the nervous system, we are not inviting the reader to picture mysterious non-natural features. All thermodynamic systems that maintain stability and integrity for the period they exist, long or short, have their own features related to their specific structural compositions and dynamic patterns. Candle flames and tornadoes are both dissipative structures that exhibit thermodynamic stability in their respective magnitudes or scales. However, only candle flames generate fast exothermic combustion reactions, radiate light, and illuminate a dark room. Conversely, tornadoes, not candle flames, can travel kilometers through large geographic areas, lifting and violently shaking heavy objects. There is nothing mysterious about these differences. They relate to each system’s respective chemical and physical features, which must be considered to explain the varied phenomena associated with each system. What is a candle doing as a system when its flame radiates light and warms up our hands? From the systemic thermodynamic point of view, it is simply maintaining its stability and integrity as a dissipative system. When a tornado passes through a village and destroys the houses, what is it doing as a system? Again, from the systemic thermodynamic point of view, it is simply maintaining its stability and integrity as a dissipative system. But, if both systems are doing the same, how do they generate such different phenomena and results? The answer lies in the unique features of each system, the context in which they form, their material qualities, and so on.

The nervous system is a homeostatic energy system. Still, the specific way it manifests such quality given its biological (e.g., histological) composition, anatomical structure and physiological organization, its looped coupling with both the internal milieu and the external environment, its development within the organism, generate distinctive results and phenomena called behavior and cognition. In what follows, we will review the general systemic conditions that run for the nervous system.

## General Systemic Conditions

To understand the nervous system and the phenomena typically associated with its functioning (e.g., perception, motor control, language, and consciousness) it is crucial to examine its peculiarities and distinctive features as a system. However, it is equally important to consider the conditions that the nervous system shares with all natural systems, living and not-living, and according to which it must work. After all, what is fascinating about the nervous system is that, being a natural system (that is, a system that respects the laws, conditions, and principles that rule and restrict every natural system), it can generate phenomena as peculiar and exceptional as perceptual experience, understanding, consciousness, language, and intelligent reasoning.

This latter explanatory exercise is essential because, when facing extremely complex explanatory problems, it is usually tempting and easy to resort to the strategy of endowing the components and explanatory machinery of the system under study with the very special and complex properties we want to explain. For instance, this was the case with the explanation of the phenomenon of life. For an extended period, it was assumed that the components of living beings were unique in that they were endowed with a certain kind of vital force or energy that was not present in the components of inert objects (Bechtel and Richardson, 1998). We tried to explain life by postulating that the matter of which living beings are made was itself, somehow, living. Similarly, when facing the problem of explaining cognitive and mental phenomena, such as perception or intelligent reasoning, it is tempting to think of the nervous system, its components, and machinery, as if they themselves operated with protocognitive (subpersonal, automatic, unconscious) cognitive mechanisms, as if the nervous system was an epistemic agent dealing itself with alleged problems of uncertainty and lack of information, working on the base of hypotheses, inferences, predictions, error detection, and looking for evidence and hypothesis confirmation.

As the cases of biology and the problem of life teach us, the strategy of projecting the properties and capacities of the explanandum, even in a carefully sophisticated deflationary way, into the explanatory substratum itself does not lead to adequate explanations. We think we would do better if we take the nervous system not as a cognitive agent but as a physical machine (Ashby, 1947) and try to understand its operation according to the conditions that rule every physical system in general. Doing this does not mean, of course, ignoring the particular features of the nervous system regarding its structure and organization; it just means understanding that such specific features do not set the nervous system apart from the rest of the natural systems.

Before we further develop our argument for a strict naturalistic approach to explain the emergence of behavior, we consider it essential to lay out some foundational concepts, so the reader can better consider the starting points. These points are not meant to provide an exhaustive characterization of the nervous system; far from that. However, combined, they should help us understand, in broad terms, the way the nervous system operates and generates some of the phenomena associated with its functioning. We consider the following premises:

1. The nervous system is non-teleological. Its dynamics are not driven by purposes or goals. As is the case with natural systems in general, the dynamics of the nervous system unfold following physical laws that are blind to purposes or goals (Villalobos and Ward, 2015).
2. The nervous system is non-normative. Its dynamics are not based on normative considerations such as what is (or might be) good or bad, adequate, or inadequate, beneficial, or harmful to the system itself or the organism. As is the case with natural systems in general, the dynamics of the nervous system unfold following physical laws that are blind to normative values (Villalobos and Ward, 2015).
3. The interactions of the nervous system with its surrounding systems, both intra- and extra-organism, are structural (i.e., physical, chemical, energetic) in nature, not epistemic, informative, or cognitive (Maturana, 2002). The nervous system is not an epistemic agent that collects and processes information, and its functioning is not oriented to knowing (inferring, predicting, guessing) anything (Villalobos, 2015).
4. The components of the nervous system, its neurons, and networks work through strictly local interactions, without “having in view” distal states, either intra- or extra-organism (Maturana and Varela, 1987).
5. The nervous system operates in its continuous structural present, without “having in view” non-current states, either past or future (Ashby, 1960; Maturana, 2008).
6. The nervous system, at the neuroscience scale of analysis, behaves deterministically (Ashby, 1960; Maturana, 1980). It is not a free agent that chooses, among a set of possibilities, what to do. The nervous system does what it does every instant because its structure at that instant simply allows no other action.
7. The nervous system is an open thermodynamic system that exchanges matter and energy with its surroundings.
8. The nervous system is a homeostatic system that, like all homeostatic systems, maintains certain stability and equilibrium in its physical parameters and shows the capacity to restore them when they are disturbed within specific ranges (Ashby, 1960).
9. Nervous systems, since their first formation in the embryonal stage, grow and develop in the continuous coupling, adaptation, and structural coherence with their biological surroundings and the extra-organism environment. This is a trivial condition for every system. Everything that begins to exist does so because the conditions for its emergence and existence are given. Every system emerges adapted to, or in structural coherence with, its surrounding conditions. This adaptation is conserved while the system exists as such and lost when the system ceases to exist.
10. A nervous system with normal anatomical and physiological development is always coupled in a loop with:
  (i) other physiological systems of the organism, such as the endocrine, immune, cardiovascular, and digestive systems.
  (ii) the external environment through specialized sensory organs and motor structures. Since these couplings are functionally closed as feedback loops, the nervous system always affects itself through them and thus maintains its homeostasis. At the same time, since these couplings arise in structural coherence and adaptation from the beginning (recall point 9), the self-centered homeostatic dynamics of the nervous system result in the conservation of the adaptation of the rest of the organism.
11. Complex enough nervous systems are hierarchically organized as second-order homeostatic systems, therefore exhibiting ultrastability and great flexibility (Ashby, 1960). Hierarchy, in this context, implies that some of the feedback loops of the nervous system (mentioned in point 10) operate at the first level of stability, whereas others operate over them at a higher level. In this functional organization, the higher level constraints but does not eliminate the degrees of freedom of the lower level, so the latter can deploy a considerable range of variability in its dynamics to the extent that does not disturb the equilibrium of the former. Because of this, from the point of view of the higher level of homeostasis, the lower level will appear to show not only adaptive or “useful” dynamics but also “neutral” or “useless” ones.

In the following sections, we will elaborate on the EHP considering this set of premises to produce a plausible explanation for behavior and, ultimately, understanding.We will start arguing how a naturalistic approach is required to disentangle proximate causes (cell operation) from distal causes (organism operations). Then, we will build over this conception to reinterpret neural processing without goal or purpose. We will also evaluate anticipatory behavior by means of the EHP and contrast it with the FEP. Finally, we will offer an evolutionary argument regarding how these apparently goal-directed-behaviors emerge from non-teleological mechanisms. Moreover, we will discuss how useless behavior may appear and may constitute a potential adaptive advantage in evolutionary terms.

## Some Specific Considerations About The Nervous System

One way to illustrate how neuronal interactions are restricted, and therefore, locally driven interactions dynamics, is to realize their context. When comparing the whole organism to its component cells, or even organs, it can be noted that cells are sensitive to completely different scales of physical phenomena (Southern et al., 2008; Dada and Mendes, 2011). For instance, swimming in a pool or the ocean makes little difference to an experienced swimmer, whereas doing so in an aqueous solution would be lethal to a cell (Pedersen et al., 2011). This becomes very clear at the spatial and temporal scales (Engel, 1980; Southern et al., 2008; Dada and Mendes, 2011; DiFrisco, 2017). For example, at the chemical level, cells are most sensitive to their direct environment, a space in the order of micrometers or smaller, whereas we, as organisms, are sensitive to phenomena in the order of millimeters and beyond. Regarding the time scale, the difference is equally remarkable. Most of our cells are replaced in our lifetime (DiFrisco, 2017), which means their time scale is significantly shorter than ours.

We may argue the specifics of these differences, such as up to what point the scales overlap, or how arbitrary it is even to state that such scales exist. However, the core of that observation goes beyond the scales themselves, the point being the phenomenological operational closure of a whole human being compared with a single cell is remarkably different. What I see as a hamburger is not the same experience for a cell. On the one hand, a cell is too tiny to perceive the hamburger as a whole, but also its potential interactions with it are different from those we would engage in. There is a difference between how we perceive and the actions we may perform given such perception; how we couple with objects in behavior. As such, even if we would acknowledge that a neuron or neural network could foresee something, it would be in a shorter time span and based on their local interactions.

**Figure 1:**
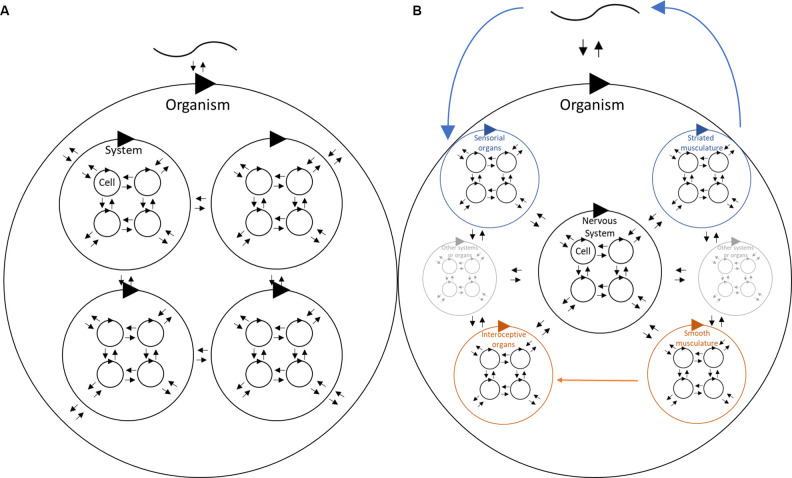
Schematic hierarchical structure and interactions for **(A)** a generic living organism, and **(B)** highlighting particular features of the nervous system. In **(A)** self-closed arrows distinguish an operation closure denoting a certain level of independence of its internal operations. Bidirectional arrows for each self-closed arrows denote the local interactions at each level (cell, system, organism), highlighting the relevance of local interactions. The cohesive and codependent interactions that lead to the emergence of a system as a recognizable unit are represented by a bigger circle denoting a self-closed arrow containing those local interactions (i.e., System, Organism). These systems may also interact with each other at a higher level (in terms of space and time) of interactions. Finally, the codependence of these systems will finally produce what we recognize as an organism following the same rationale, while then interactions are established with what we traditionally recognize as the environment (depicted by the upper curve). In the case of **(B)**, we highlighted the sensorimotor loop (internal and external), as well as the structure of sensorial structures, middle structures (traditionally referred to as processing related areas), and finally effectors. In this figure, we highlighted only muscles, but effectors cover a major range of structures, such as glands.

The global concept of how local interactions build up hierarchically to behavior is depicted in Figure 1A, where we intend to remark local interactions. For instance, cells may interact directly with other cellular phenomena only. By doing so, they are structurally coupled with the environment, and if alive, maintain their energetic equilibrium and, therefore, their operational closure (close-loop arrow). Hierarchically, these local interactions may lead to population phenomena, such as synchronization. Given the intricate codependence between the actions of individual cells, a group of cells starts to behave as a unity, like a fish shoal showing coordinated movements (Herbert-Read, 2016), or eusocial insects, where survival is a matter of the colony and not only of the individual (Gillooly et al., 2010). In both examples, there are not unique individuals signaling what has to be done to the colony, but rather local interactions as one-to-one individuals produce these complex phenomena. For instance, the fish shoal seems to move like a wholly coordinated system, while this global property answers to individual interactions of one fish considering the movements of the fish right next to it (Herbert-Read et al., 2011). As such, complex systemic phenomena may occur driven by local interactions when sensorimotor actions of individual entities are codependent and intimately coupled (Bonabeau et al., 1997). This distinction is critical to avoid extrapolating system properties to local components; however, it raises some challenges. Given our aim to explain the emergence of behavior from a naturalistic viewpoint, the difference in sensitivity is challenging for at least two reasons. The first reason is the difficulty in establishing relationships between these levels; if they do not perceive the same phenomena, how are their dynamics aligned for survival? This complicates the development of causal explanations in biology. A similar situation was noticed 60 years ago by Ernst Mayr (1961) when he established that virtually all explanations of biological phenomena consisted of sets of proximate causes and sets of ultimate causes (or distal, given our framework). In Ernst Mayr’s work, he illustrates the difficulties in establishing the causes of behavior, arguing that they can be attributed to the environment, physiology (including molecular mechanisms), or the interaction between the two. In this context, proximate causes would be those that control the organism’s responses to immediate environmental factors (such as the sunrise regulation of the sleep-wake cycle in a mouse), while ultimate causes would be those that have an impact on the organism’s survival (such as increased nocturnal activity in mice that decreases the probability of encountering predators). These ultimate causes are rooted in evolutionary mechanisms and have been incorporated into the system through generations of natural selection (Mayr, 1961). Therefore, under the EHP view, behavior emerges from the intersection of coupled local interactions, which keep cells alive, and evolutionary pressure, that permits local conditions to remain coupled, if they do not jeopardize the life of the whole organism (the distal cause). It is critical to notice that the distal cause can be interpreted as a consequence of meeting local requirements. Recalling point 6, “The nervous system does what it does at every instant because its structure at that instant simply allows no other action”. In other words, distal causes exist as a result of living beings staying alive coupled with their environment and restricted to the evolutionary and individual history that has determined particular properties of their structure.

There is a second reason where local interactions are relevant. For the organism to survive, the fundamental needs of all these hierarchic levels must be met (Figure 1A). The specific needs of different kinds of cells are varied and different from those of the organisms they compose. Therefore, there are multiple layers or levels of operational closure that are not strictly equivalent nor overlapped and they must meet the entire organism requirements to stay alive and coupled with its environment. This illustrates the complex synchronization that must occur in the cell population of such an organism to survive, as well as the close codependence of a variety of cellular populations with remarkably different requirements.

Now, an apparent contradiction appears. Despite the short overlap of sensitivity to phenomena between the parts of our body and the whole organism, we exhibit adaptative behaviors. This supposed paradox has been solved mainly by assigning functions aimed at the survival of the entire organism to different parts of the body (Roux, 2014). However, this position usually omits the evolutionary process that led to those functions, while also neglecting the survival of the cells that live in the organism. It is critical to note that many of our cells die each day and that each of these cells has different survival requirements and may not act in alignment with the survival of the whole organism. This is evident in pathologies such as cancer (Chaffer and Weinberg, 2011) and autoimmune diseases (Park and Kupper, 2015). We tend to refer to these conditions as errors or problems of specific systems and functions, overlooking that, since cells live in us, but not for us, there is a possibility that these phenomena may occur. As far as the global system (i.e., organism) meets its requirements, codependence relations will keep the system alive, regardless of other local interactions with no adaptive nor maladaptive values that may emerge.

Our alternative approach would be to consider that each cell meets its own requirements to survive. In this sense, it is essential to assume that the cell, as an autopoietic unit, can respond and exert control over its niche, but only within its local environment. Thus, specific environmental conditions that occur in localized regions of our body will set in motion different cellular mechanisms. Since cells can only directly influence that local environment, they can only meet their requirements. Naturally, these local interactions may have distal impacts (as Ernst Mayr conception); most of the time, when all cells meet their requirements, they indirectly end up meeting ours. As such, behavior can be considered an emergent property derived from the individual actions of cells that lead to their survival, and ultimately to ours. These two levels must be aligned for the whole organism to survive; however, there is a possibility of mismatch where some are neutral (without significant consequences) while others give rise to what we call pathology.

This different approach can be described as an interaction of parallel causes and requirements nested in cells and organisms, in the sense that the phenomena present in individual cells mirror a distal effect on the whole organism and* vice versa*. Therefore, we may explain behavior from the viewpoint of the entire organism or the interactions of its cells. However, a more comprehensive approach would be to track cellular interactions up to the mirrored effect on the organisms without neglecting that the proximal causes affecting each layer or level are aligned for survival. As such, the same phenomenon can present a different impact on the organism and the cell populations within. For instance, covering the head with the limbs to block a hit to the head is adaptive for organisms, yet limb cells will die as a result, and the behavior would not be adaptive for them.

At this point, we may start asking which is the most relevant aspect of cells’ survival. Naturally, energy management is critical for survival in any cell, as they must balance expenditure with income and maintain an adequate reserve to cope with environmental restrictions. If we consider a cell that lives within an organism and that has an evident impact over its behavior, such as a neuron, this premise stands. For neurons to survive, they must properly manage their energy budget. Problems of the organism, such as avoiding injuries, coupling with stressful work, dealing with the death of a close one, and so on, are not part of the proximal phenomena stressing a single neuron. Of course, those phenomena have stimuli transduction into local neural requirements: energy demand imposition. Therefore, neurons will deploy mechanisms to couple with their local requirements and, hopefully, they will solve the organism’s problems as well. As such, when describing how behavior emerges, we should always map the differences between the organism and cellular domain of interactions. For instance, Vergara et al. (2019) described perceptual stimuli as mapped into physiology with different impacts at each level. An organism may just be looking at something. At the same time, transduction sets electromagnetic waves of the visual spectrum into action potentials, which in turn produce a cascade effect all over the nervous system, impacting the energetic demands of neurons and glia (Vergara et al., 2019). Depending on how demanding this stimulus is energetically, neurons may regulate their synaptic weights (Barral and Reyes, 2016), producing a new functional network. This new functional network will, in turn, activate muscles leading to visible behavior that may change the stimulus (e.g., closing the eyes).

This rationale is what we depict in Figure 1B, where we remark the particular conditions of the nervous system. All sensory inputs, driven from sensory organs, internal or external, are activated by stimulation that impose energetic demands on the nervous system. The system can affect that energy imposition by effector activity, such as muscle activation, among others. As such, closing the eyes will reduce the amount of spent energy driven by visual perception. It is also relevant to notice that for a single neuron, or even for a central nervous system neural network, it makes almost no difference if the signal arrives from interoceptive receptors or perception organs. The stimulation received is, in physical and chemical terms, the same. However, as previously implied in point 11, the feedback loops established by the nervous system through perceptual and interoceptive structures are hierarchically organized in such a way that their respective dynamics get coordinated. Also, we must not forget that the organisms not only interact with the environment through the nervous system, and that the nervous system is also coupled with other physiological systems, obeying the same rationale of local interactions.

In this framework, the energy balance mechanisms of the cells have a consequential impact on physiology resulting in the emergence of behavior. At the same time, since the cellular and whole-organism levels are analogous to nested layers or levels, the behavior itself will impact not only the experience of the whole organism but also the cells that compose it. It may be the case that only some of the cells are affected, which remarks the need of recognizing that the same phenomena may impact differently the whole organism and regions (cells) within. Also relevant is the fact that neurons cannot directly experience the stimuli that trigger organism behaviors. Once sensorial transduction is made, only proximal phenomena such as action potential, lactate transporter activation, synaptic modulations, and so on, are observable. In other words, cells such as neurons are never solving a mathematical problem, or recognizing a face, but are only solving energy needs required for their survival.

## Reinterpreting Neural Processing

The notion that behavior is not inside the machine is notably exemplified in the experiments in “synthetic psychology” of Braitenberg (1986). He presented how simple mechanisms may lead to complex behaviors and the illusion of complex cognitive processing. The complexity may be loaned from the environment, while internal mechanisms can stay simple. We usually think of neural mechanisms as complex and difficult to assess, based on the complexity of behavior. Let us assume for a moment that it might be the case that neural mechanisms are relatively simple and that most of the complexity we see in our behavior is loaned from our environment. Is there an experiment like Braitenberg’s, in which we can test real neurons?

Novellino et al. (2007) and Tessadori et al. (2013) presented an experiment resembling Braitenberg’s vehicles using neuron cultures (actual neurons, not artificial neural networks). In this setup, a cart decodes distance to objects using a firing rate paradigm, and then the same paradigm is used to code back the wheels’ speed independently. If the cart crashes, a stimulation burst of 20 Hz for 2 s is delivered (Tessadori et al., 2013). Under this protocol, the neuron culture learns to avoid obstacles. Thus, as external observers, we may be tempted to say that the cart does not like to crash, and it, therefore, learns to avoid obstacles. Even more, we are tempted to say that the goal of such behavior is to avoid crashes. However, that stimulation pattern is known to trigger plasticity (Madhavan et al., 2007; Chiappalone et al., 2008; le Feber et al., 2010). We may also argue that each time the cart crashes, it induces plasticity, changing the functional network. Considering how the experiment is set up, the changes will keep occurring unless crashes are avoided. Once no more crashes occur, no more changes in the network are expected. In other words, a functional neural network will keep changing until an “obstacle avoidance” structure emerges, and we will be tempted to say that the neural culture learned to avoid obstacles.

Critically, the functional network does not appear by means of an impact-avoidance goal, but as an effect derived from the energy demands posed by the stimulation that drives plasticity. Our proximate cause was energy demands, while the distal effect was avoiding obstacles. Importantly, this effect is structurally determined by how the wiring and stimulation conditions were set to the vehicle controlled by the neuron culture, meaning that a wider set of “learnings” can emerge if the structure changes. Under this framework, it is rather useless to think that, at the neuron level, a particular neuron or set of neurons are “processing obstacle avoidance”, or that there is an obstacle avoidance network in the neuronal culture. At the level of the organism, we can be tempted to use this approach, and it might be even helpful in some contexts. Nonetheless, to explain how the vehicle learns, we must consider that individual neurons deal with significant energy demands that trigger plasticity as a compensation mechanism (Vergara et al., 2019), which produces the avoidance of obstacles as emergent behavior.

**Figure 2:**
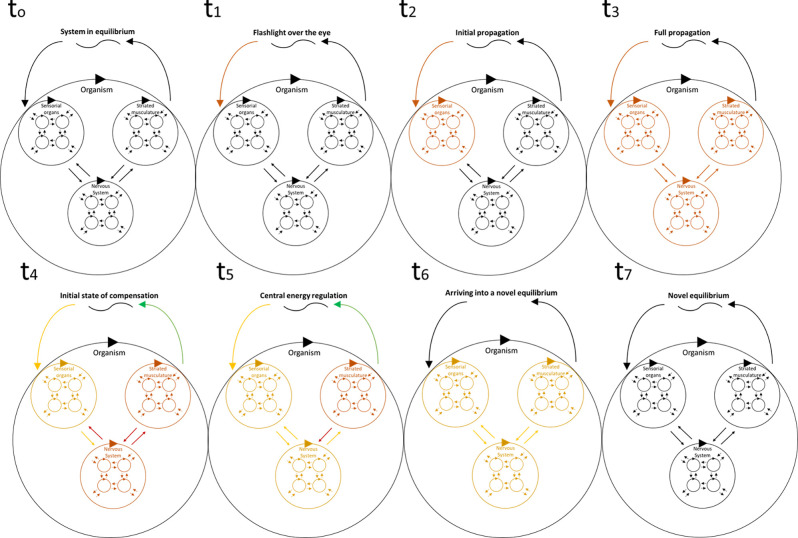
A simplified version of Figure 1B depicting temporal dynamics driven by external stimuli that push the system out of its energetic equilibrium. This figure does not intend to be an exhaustive description of the temporal dynamics of this process, rather to depict some critical features of such dynamics. In particular, that the energy demand imposition of the initial stimulus would only be detected by the sensory regions, and then propagate into the remaining areas. We also emphasize that these regions are bidirectionally related, so that, before the behavior occurs, it is expected that some energy compensations of single neurons and neural networks will take place. When the system is in full propagation of the energy demand imposed by the environment, a behavior will take place impacting the perceived environment. This will have an effect on the sensory areas. However, the impact is not immediate, and it will return to a propagation process in which the mechanisms of all levels (neuron, neural networks, organism) will find a novel equilibrium.

It is possible to establish that, in proximal terms, neurons must efficiently solve their energy management. As depicted in Figure 2, we expect that a neural network in equilibrium will lose its energy balance driven by external stimuli. The energy imbalance will propagate through the network according to its structural constraints. Since most neural connections with different regions are bidirectional, the system will generate a global answer (as observers, we may declare it a coordinated answer). Eventually, this will get to the effectors (full propagation is achieved). At that moment, the organism will be able to take action as a whole system to impact the input stimulus that has disturbed the energy balance. It is critical to notice that, in the meantime, local mechanisms of single neurons are triggered to couple with this increment in energy demand as well. Within this close-loop structure, the actions taken by the entire organism, as well as those taken by individual cells, will allow a new energy equilibrium to be achieved, which will be a novel functional neural structure associated with a novel behavior.

From the previous argument highlighting energy as a key regulatory element, makes sense considering neurons’ proximal context as the trigger of neuron regulation. Neurons are extremely sensitive to oxygen deprivation (Ames, 2000) and the central nervous system possesses small glycogen reserves (Brown and Ransom, 2007). Neurons answer to energy demands (neural activity) by outsourcing their energy needs to the glia (Weber and Barros, 2015), which will trigger the neurovascular coupling associated with neural activity (Sokoloff, 2008; Schulz et al., 2012; Robinson and Jackson, 2016), followed by increased glucose uptake and glycolytic rate of astrocytes (Magistretti and Allaman, 2018). In addition, neuronal mitochondria increase ATP synthesis in response to an increment in synaptic stimuli (Jekabsons and Nicholls, 2004; Connolly et al., 2014; Rangaraju et al., 2014; Toloe et al., 2014; Lange et al., 2015). These are just the early responses in the range of hours, as the synaptic scaling ends balancing to a homeostatic level of neurons’ activity (Barral and Reyes, 2016), reducing the energy cost of the activity increment. An increment in stimulation is expected to produce long-term network modularization (Novellino et al., 2007). Interestingly enough, when significant downscaling occurs, a few synaptic weights (dendritic spines) will increase (El-Boustani et al., 2018; Jungenitz et al., 2018). As such, while we have only described the proximal actions of neurons, they have a vast impact on the neural networks and therefore behavior. It is plausible to observe neural processing as an emergent property rooted in proximal cells requirements.

Up to this point, we have been able to rephrase neural processing without purpose or so-called “goal-oriented behaviors”. Our explanation has also been faithful to a structural determinism, meaning that behavior in the vehicle (i.e., neuronal culture) emerges as a result of neurons doing the actions determined by their properties and structure. So far, introducing volition or desires in this context would be to acknowledge openly that a culture of neurons shares the same properties we usually attribute to a whole organism. However, does this reinterpretation lead to new implications?

The most obvious is the reinterpretation of key phenomena into local interactions. For instance, neural activity, usually seen as neural processing, would be interpreted as energy expenditure, as an environmental pressure for a neuron, which forces it to activate mechanisms to balance its energy budget. Otherwise, it dies. Plasticity, classically viewed as a learning mechanism (please note how a molecular mechanism has a whole system property; learning), would be reinterpreted as a coupling mechanism of neurons to deal with incoming energetic demands from presynaptic neurons. As stated in Vergara et al. (2019), the synaptic gain will change to match a homeostatic energy level. This immediately sets some empirical implications. For instance, synaptic scaling should answer to stimulation, but also to energy availability. Therefore, changes in glucose availability in a neuronal culture should change the dynamics of classic synaptic scaling protocols. Specifically, synaptic scaling should be higher in the case of less glucose availability (for more empirical predictions, see Vergara et al., 2019).

Another consequence of viewing neuronal processing as an emergent property of individual neurons displaying mechanisms that allow them to stay alive under different energy pressures is that not everything neurons do is helpful to the organism. In other words, since neurons are only solving their local requirements, their actions may lead to the emergence of useless behaviors. This means that part of the neural network activity, which can respond to the continuous activity of multiple stimuli, will lead to the appearance of behaviors with no apparent usefulness and that may even be maladaptive. This is necessarily the consequence of a codependent system governed by local actions. Each component solves its requirements as part of its condition of existence, but once they are solved, other harmless actions may occur as a kind of debris that results from the operational closure. It is crucial to notice that as living beings we do not need a perfect functional coupling with the environment; it must be just good enough to survive. If we consider further the hierarchic structure, even relevant actions for survival at a single cell level may have useless or undesired impacts at the whole-organism level. As long as survival is not immediately compromised, e.g., as far as physiologically critical homeostatic systems are not driven away from equilibrium, these mismatches may freely occur. This consequence frees us from the need to include a function in every behavior we have. Many of them can be helpful for our survival and others may not, but above all, given the degree of freedom allowed by the hierarchical organization of the neural-body-environment homeostatic mechanisms, we can have neutral behaviors from an adaptative viewpoint.

This last point is critical since the degree of behavioral flexibility increases the probability of producing neutral behaviors and deleterious ones. Thus, it is not surprising that animals with high behavioral flexibility are associated with greater effort and parenting times during ontogeny (Isler and van Schaik, 2009, 2012; Barton and Capellini, 2011; Heldstab et al., 2019; Uomini et al., 2020). One only needs to observe how a toddler relates to its environment to discover that many of our behaviors during infancy put our survival or fitness at peril. Parental care or parenting allows us to buffer this flexibility, allowing us to stay alive. Conversely, flexibility also allows us to increase fitness by adapting to the environment during ontogeny, unlike less flexible animals requiring phylogenetic mechanisms of change to adapt.

## Building Up to Complex Behaviors

Behavioral flexibility by means of EHP is a powerful concept, as it explains fast changes in behavior during ontogeny, but it also allows the test-retest rationale to operate. As far as the test-retest rationale follows the restrictions imposed by single-cell energy management, learning can emerge. We expect that this flexibility is what ultimately gives rise to the most complex cognitive phenomena, such as understanding. Specifically, what we refer to as useless behavior can be interpreted out of the teleological paradigm as behavioral flexibility. Those apparently useless behaviors may find their usefulness when an environmental pressure is relieved by this behavior, or they may never find their usefulness from the observer’s position. From a naturalistic approach, this is just flexibility to couple with the environment following point 8, describing the nervous system as a homeostatic system that will maintain certain stability and equilibrium and restore it to a certain extent.

In this view, complex cognitive phenomena emerge from this hierarchical flexibility of the system. These more sophisticated cognitive phenomena are vastly discussed and modeled using the Free Energy Principle (Friston, 2010). How does EHP stand in contrast to FEP? The FEP is an organism-based approach that considers volition as a critical element, especially when regarding aspects such as understanding (Yufik and Friston, 2016), as it distinguishes lower forms of learning, allowing the introduction of cognitive models. Therefore, as an initial difference, we noticed that FEP rather omits neuron requirements, assuming them as chronically met. Secondly, it assumes the presence of goal-oriented behaviors, volition, and purpose, which is to be expected if starting from a whole organism viewpoint.

Although there are obvious differences between these two perspectives, especially since the FEP contains teleological elements and considers the nervous system as an epistemic agent (points 1 and 3 of “General Systemic Conditions” section), Yufik’s proposal (Yufik, 2013, 2019; Yufik and Friston, 2016) is very similar to the EHP at the neural network level. He developed the idea of how neural assemblies (or packets) would appear, producing functional networks which allow understanding to emerge. The core idea envisions the mind as a cartographer mapping the environment, similar to classic cognitive perspectives (Bateson, 2015), where modularization of functional neural activity will allow differences to be made. In order to establish that two objects are different, a difference in the functional network should emerge (different packets or sets of packets) to allow the recognition of such distinction. Following our rationale, the critical question is what local mechanism is driving the emergence of those distinctions. During the works of this thermodynamic conception of cognition, there is an acknowledgment of the relevance of energy in modulating the packets’ emergence in this proposal (Yufik, 2013, 2019; Yufik and Friston, 2016). For instance, cortical tone (temperature of this thermodynamic formalization), which can be rephrased as energy demands using EHP, is critical in how the system will react towards the equilibrium by FEP conception (Yufik, 2013).

This is a critical aspect, as in this FEP-driven proposal energy conditions modify the neural functional structure to produce a novel equilibrium. This conceptually very similar to the EHP, as depicted in Figure 2, achieves a novel equilibrium by a new energetic demand (i.e., cortical tone). Even more interesting is that in Yufik’s work (Yufik, 2013, 2019; Yufik and Friston, 2016) modularization is expected from a learning process, the same process reported by Tessadori et al. (2013) and Novellino et al. (2007), which we have explained from an EHP viewpoint above. This role of energy management is even more explicit in the following communications (Yufik, 2019), mainly focused on the demand or energy expenditure and availability. Therefore, both approaches find common ground in the middle, acknowledging that local neuron requirements (i.e., energy management) are critical for modularization to occur, leading to cognitive distinctions that will ultimately produce understanding.

It is relevant to notice that EHP and FEP are two sides of the same coin. Following the parallel conception of organism vs. cell community approach, all conceptions derived from FEP could be mapped in EHP terms and *vice versa*. Naturally, as we get closer to cellular processes, FEP is less precise on its implications, and when getting closer to high cognitive functions, EHP is rather vague. However, reasonable efforts can be made to understand what is happening at cellular and physiological levels when we describe the cognitive mechanism. For instance, one challenging explanation to be made from the EHP side is anticipatory behavior. How can neurons caring about their local needs solve upcoming organism events?

One key aspect of anticipatory behavior is that it must be learned first. In other words, it is not anticipating anything, it is rather re-evoking structural history. This means that most predictions we make are based upon past experiences. Therefore, we avoid pain, as we have previously experienced pain. Similar to what we described in Tessadori’s vehicle case (Tessadori et al., 2013), energy demands derived from the painful stimulation lead to restructuration, allowing pain avoidance to occur (rephrased as reducing surprise by FEP means). If we focused not on the result but on the learning phase, we would notice that consistent unrelated stimulus (e.g., a light turning on, an acoustic event, or a similar signal event) is followed by pain.

Light, sound, and pain produce energy demands through perception. Nonetheless, the pain has a durable effect, which means a long-lasting energy demand situation. Also, its intensity is directly related to the amount of damage (Dubin and Patapoutian, 2010). Therefore, that is the critical stimulation to be avoided by means of local neuron requirements.

When we focus on neural activity during situations of these characteristics, we observe that both neural activities, the one derived from the upcoming pain signal and the one directly derived from pain, begin to fire closer in time through learning (Urien et al., 2018). The overall activity appears to be the same, but the temporal aspect change. Basically, now the signal triggers both the signal-related activity and the originally pain-driven avoidance behavior. The critical aspect here is that the signal that anticipates pain does not mean pain itself, but in neural activity, the signal packet (assembly) will fire just before the avoidance behavior packet. Following the logic of *fire together, wire together*, the avoidance packet will ultimately be activated without the pain but with the signal packet, meaning the fusion of these two packets. Please note that this explanation does not involve mental manipulations yet as the ones suggested by FEP, and we can still be faithful to our premises.

From EHP, the fusion of these neural activities into one module that would lead to the so-called anticipatory behavior, is driven by the same rationale observed in Figure 2. Basically, the initial trial will deploy many behaviors that will not be useful to keep the equilibrium, while at the same time the propagation of the energy demand imposed by pain will, in consequence, functionally restructure the network with each iteration. Following the same proposed mechanism for the vehicle controlled by a neuronal culture, at some point behavior will satisfy the condition of approaching neurons to a novel equilibrium. During this central energy regulation, with each iteration the best “pain-avoidance” structure will be selected until the predictive behavior is settled. These changes may even follow a random structure change, and they would still work. However, neural mechanisms such as synaptic reinforcement by fire together, wire together (Abbott and Nelson, 2000), play a critical role for this to happen efficiently. Considering that the EHP reinterprets these plastic mechanisms as coping energy mechanisms of neurons, we are able to explain these phenomena without yet needing to call for complex mental scenarios. Naturally, this explanation does not cover more sophisticated behaviors like planning, which under a classic view require volitional manipulation of information. However, it sheds light on how, starting from cellular communities, “goal-directed” behaviors can be explained leaving the goal as the consequence, not the cause. Neurons don’t even realize that the animal was submitted to pain; they just react according to their local requirements. It is we who, as observers, are tempted to say that the animal learns to anticipate the aversive stimulus. Even more relevant is the fact that as we show anticipatory behavior, we may be blind to the actual causes that led to this apparent anticipatory behavior by neglecting history, which under the EHP view is no more than an expression of an organism coupled with its environment where its particular history defines the behaviors that will be deployed when observing the signal related to pain.

Under this context, we have given an explanation of how an organism can act in the prediction of hazard, without actually predicting it. Local neural properties allow these phenomena to occur without incorporating purpose, mental model, or further mental scenarios. Notably, FEP and EHP, despite their differences in starting points (and, therefore, conceptual frameworks), share similar predictions on how neural networks would operate. Distinctions are made on what produces those changes. Another relevant difference of our approach is that neurons can fulfill their requirements without solving the problem of the whole organism but never endangering the life of the organism (at least not immediately). Therefore, the behavioral flexibility given by the impact left by neurons when solving their needs could have a negative, neutral, or positive impact, which means that the neurons may find local energy homeostasis attractors that satisfy their requirements but not necessarily the organism’s requirements. However, if so, why does it seem that they are almost always positive (hence the teleological need to indicate their function)?

## An Evolutionary Perspective on The Coupling of Different Levels of Operational Closures

We see what remains, not what has been. During the evolutionary history of living beings, most species have disappeared, have become extinct (Newman, 1997). In fact, the species that are alive today represent less than 1% of the historical total (Newman, 1997; Jablonski, 2004). This makes it risky to use evidence only from modern animals to explain the relationship between the cellular and whole-organism levels of organization. On the other hand, virtually all present-day animal body plans date at least back to the Cambrian explosion (CE), an event that occurred more than 500 million years ago (Maloof et al., 2010). While it is still a matter of debate, it is possible to propose that near that time window, a level of animal diversification and radiation occurred that had not been seen before and has not been seen since (Keijzer, 2015; Trestman, 2013).

Interestingly, this period has also been ascribed as when metazoans with complex active bodies appeared (Trestman, 2013). These organisms are defined by having: (i) articulated appendages; (ii) many degrees of freedom of controlled movement; (iii) true senses (with specialized organs such as eyes); (iv) sense-guided motility; and (v) anatomical capacity for object manipulation (Trestman, 2013). The appearance of metazoans probably occurred at least 200 million years before the CE (Erwin, 2015; Dohrmann and Wörheide, 2017), and the nervous system probably appeared during the Ediacaran period (635 million years ago). In simpler metazoans with low-complexity nervous systems, synchrony between the neuronal and organism levels was probably much easier to achieve than in animals with complex active bodies. Movement is not yet a problem for those animals. Thus, it is feasible that, during the initial evolution of the nervous system, a limiting element was the alignment between the neuronal level and that of the whole organism. Once this occurred, the space for possible radiation and diversification opened up.

In ontogenetic terms, the reality is similar. In animals, the highest mortality rates are usually seen early in life (Caughley, 1966), when their individual-environment relationships are still being established and they tend to have much more behavioral flexibility. Even in our species, this reality is not far off, for it has not been long since most of our offspring died during the first 3 years of life (Volk and Atkinson, 2013). The problem lies in that we often only consider its present condition when observing an organism such as ourselves and its direct relationship with the environment, ignoring its phylogenetic and ontogenetic history. Under this perspective, most cellular phenomena are aligned with their whole-organism functions. This may lead to the interpretation that the proportion of misaligned events between these levels of the organization is negligible or almost nonexistent. Thus, we only see what has worked for survival, while counterexamples of instances where cellular phenomena are misaligned with organisms vanish. In other words, under this view, we are incurring a survival bias, where we focus only on the instances where cellular and whole-organism levels overcame a selection process and overlook those that did not. This can lead us to false conclusions, such as overrepresenting aligned states or assuming cellular levels have functions for our survival.

This also translates into a tradeoff between flexibility and survivability. Higher degrees of freedom and higher levels of flexibility allow the emergence of novel adaptations, which increase the organism’s fitness. This context can also explain why larger nervous systems (brains with more neurons) are associated with greater behavioral richness. A larger number of neurons leads to a greater diversity of local responses/solutions and greater behavioral flexibility. However, on the other hand, there may be a maximum of possible degrees of freedom before the number of misalignments between cellular and whole-organism levels can remain functional.

**Figure 3:**
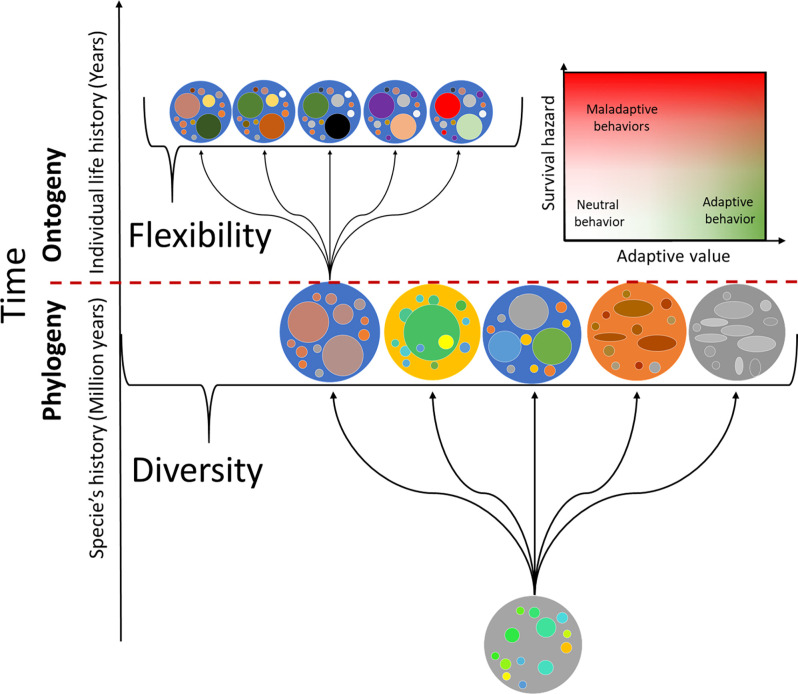
Schematic of the historic progression of organism coupling with their environment leading to a diversity of organisms (phylogenetic mechanisms) and behavioral flexibility (ontogenetic mechanisms). Evolution may generate important changes in organism morphology, producing major differences between different species (depicted with different shapes and colors). Ontogenetic mechanisms will be constrained by phylogenetic history but will produce a large number of potential behaviors from which only a subsample will appear. The behaviors that appear during ontogeny will depend on the individual life history of each organism that will lead iteratively to different functional structures (depicted as change in color but not shape), which will ultimately produce different behaviors and functional constraints.

Another point to consider is that not necessarily every lack of synchrony is maladaptive. There is the possibility that some of the neuronal activity that is not fully aligned with the organism is “neutral.” Thus, analogous to models of neutral evolution, it is feasible that a non-trivial proportion of what neurons do to solve their local energy requirements has no significant impact on the organism’s survival. It is possible to postulate that the less fundamental to survival a behavior is, the more neutral activity there is. That is, the less essential behaviors probably allow for less alignment between levels. This, in turn, would increase the presence of behavioral richness or “polymorphisms” in those behaviors. Specifically, when both the adaptive value and the survival hazard are low, neutral behavior emerges (Figure 3).

Finally, it is critical to realize that, under this notion, behaviors are not goal-oriented *per se*. Many may appear as goal-directed, as they are conditions of existence of the system (e.g., breathing). Under our scope, breathing organisms stay alive, therefore exist. However, breathing was never designed or deliberately addressed to meet the oxygen requirements of the organism. When we remove the goal rationale of structures and behavior, the evolutive process in which behavior emerges loses its need for teleological explanation. As such, the brain or areas within were not designed to solve specific problems. Instead, in meeting their own requirements, cells satisfy the organism’s requirements too; if not, survival is compromised. When most cells living in the cellular community meet their requirements, the organisms will do so. It is simply the condition of existence of such a community. Behavioral diversity and flexibility emerge within these messy interactions of individual cells acting locally and producing distal effects that may not even affect them directly.

## Final Remarks

When we observe a single cell acting in an anticipatory fashion (e.g., Shirakawa, 2006), we avoid attributing it to a sense of volition, or any epistemic or informational operation. We focus on its local mechanisms which result in such anticipatory behavior. Avoiding it is reasonable, as including it obscures the mechanisms, and we also recognize the cell as a physical system determined by the mechanisms governing it. For some reason, when coming to human beings, we fail to recognize them in such a way. This is so dramatic, that besides EHP, we have no knowledge of another integrative explicative proposal of behavior using a strict naturalistic approach.

FEP is probably the most sophisticated and flexible proposal explaining human behavior as an integrative framework. However, it uses a strong epistemic rationale to explain behavior. This leads to assigning volition to all living beings (or even dissipative systems) or stating that the concept is only applicable for certain systems such as human beings. Despite the differences, it is notable that the phenomena described at the neural network level are quite similar in both proposals, meaning that both recognize more or less the same events as relevant to explain behavior. The causes of those events are different depending on which proposal framework is used.

We understand that intending to explain behavior and most sophisticated forms of it, such as understanding, is a major challenge for EHP. However, we consider that it is a required academic exercise in our current framework of neuroscience. As we have stated above, goals can easily emerge as observer assignation once the system is coupled with its environment, but from an evolutionary perspective, adaptations do not appear to solve a problem; they just appear, and they are preserved due to advantageous (or at least non-deleterious) impacts. In other words, focusing on the goal may obscure the actual mechanisms that produce the phenomena we look forward to understanding.

## Data Availability Statement

The original contributions presented in the study are included in the article, further inquiries can be directed to the corresponding author.

## Author Contributions

SV-J, MV, and RV developed the initial general argument. All authors contributed to all the drafts; nonetheless, each author did special contributions to different sections. MV formalized the general systemic conditions. RV contributed in the neural processing and building up to behavior sections. SV-J developed the evolutionary perspective. RV and PM edited the final version. All authors contributed to the article and approved the submitted version.

## Conflict of Interest

The authors declare that the research was conducted in the absence of any commercial or financial relationships that could be construed as a potential conflict of interest.

## Publisher’s Note

All claims expressed in this article are solely those of the authors and do not necessarily represent those of their affiliated organizations, or those of the publisher, the editors and the reviewers. Any product that may be evaluated in this article, or claim that may be made by its manufacturer, is not guaranteed or endorsed by the publisher.

## References

[B1] Abbott L. F. Nelson S. B. (2000). Synaptic plasticity: taming the beast. Nat. Neurosci. 3, 1178–1183. 10.1038/81453 11127835

[B2] Ames A. 3rd (2000). CNS energy metabolism as related to function. Brain Res. Rev. 34, 42–68. 10.1016/s0165-0173(00)00038-2 11086186

[B3] Ashby W. R. (1947). The nervous system as physical machine; with special reference to the origin of adaptive behaviour. Mind 56, 44–59. 10.1093/mind/lvi.221.44 20285973

[B4] Ashby W. R. (1960). Design for a Brain, 2nd edition. London: Chapman & Hall.

[B5] Barral J. Reyes A. D. (2016). Synaptic scaling rule preserves excitatory-inhibitory balance and salient neuronal network dynamics. Nat. Neurosci. 19, 1690–1696. 10.1038/nn.4415 27749827

[B6] Barton R. A. Capellini I. (2011). Maternal investment, life histories and the costs of brain growth in mammals. Proc. Natl. Acad. Sci. U S A 108, 6169–6174. 10.1073/pnas.1019140108 21444808 PMC3076843

[B7] Bateson G. (2015). Form, substance and difference. ETC: A Review of General Semantics 72, 90–104. Available online at: http://www.jstor.org/stable/24761998.

[B8] Bechtel W. Richardson R. C. (1998). “Vitalism,” in Routledge Encyclopedia of Philosophy, ed Craig E. (London: Routledge), 639–643.

[B9] Bonabeau E. Theraulaz G. Deneubourg J. L. Aron S. Camazine S. (1997). Self-organization in social insects. Trends Ecol. Evol. 12, 188–193. 10.1016/s0169-5347(97)01048-3 21238030

[B10] Braitenberg V. (1986). Vehicles: Experiments in Synthetic Psychology. Cambridge, MA: MIT Press.

[B11] Brown A. M. Ransom B. R. (2007). Astrocyte glycogen and brain energy metabolism. Glia 55, 1263–1271. 10.1002/glia.20557 17659525

[B12] Caughley G. (1966). Mortality patterns in mammals. Ecology 47, 906–918. 10.2307/1935638

[B13] Chaffer C. L. Weinberg R. A. (2011). A perspective on cancer cell metastasis. Science 331, 1559–1564. 10.1126/science.1203543 21436443

[B14] Chiappalone M. Massobrio P. Martinoia S. (2008). Network plasticity in cortical assemblies. Eur. J. Neurosci. 28, 221–237. 10.1111/j.1460-9568.2008.06259.x 18662344

[B15] Connolly N. M. C. Dussmann H. Anilkumar U. Huber H. J. Prehn J. H. M. (2014). Single-cell imaging of bioenergetic responses to neuronal excitotoxicity and oxygen and glucose deprivation. J. Neurosci. 34, 10192–10205. 10.1523/JNEUROSCI.3127-13.2014 25080581 PMC6608276

[B16] Dada J. O. Mendes P. (2011). Multi-scale modelling and simulation in systems biology. Integr. Biol. 3, 86–96. 10.1039/c0ib00075b 21212881

[B17] DiFrisco J. (2017). Time scales and levels of organization. Erkenntnis 82, 795–818. 10.1007/s10670-016-9844-4

[B18] Dohrmann M. Wörheide G. (2017). Dating early animal evolution using phylogenomic data. Sci. Rep. 7:3599. 10.1038/s41598-017-03791-w 28620233 PMC5472626

[B19] Dubin A. E. Patapoutian A. (2010). Nociceptors: the sensors of the pain pathway. J. Clin. Invest. 120, 3760–3772. 10.1172/JCI42843 21041958 PMC2964977

[B20] El-Boustani S. Ip J. P. K. Breton-Provencher V. Knott G. W. Okuno H. Bito H.. (2018). Locally coordinated synaptic plasticity of visual cortex neurons *in vivo*. Science 360, 1349–1354. 10.1126/science.aao0862 29930137 PMC6366621

[B21] Engel G. L. (1980). The clinical application of the biopsychosocial model. Am. J. Psychiatry 137, 535–544. 10.1176/ajp.137.5.535 7369396

[B22] Erwin D. H. (2015). Early metazoan life: divergence, environment and ecology. Philos. Trans. R. Soc. B Biol. Sci. 370:20150036. 10.1098/rstb.2015.0036 26554036 PMC4650120

[B23] Friston K. (2010). The free-energy principle: a unified brain theory? Nat. Rev. Neurosci. 11, 127–138. 10.1038/nrn2787 20068583

[B24] Friston K. J. Stephan K. E. (2007). Free-energy and the brain. Synthese 159, 417–458. 10.1007/s11229-007-9237-y 19325932 PMC2660582

[B25] Gillooly J. F. Hou C. Kaspari M. (2010). Eusocial insects as superorganisms: insights from metabolic theory. Commun. Integr. Biol. 3, 360–362. 10.4161/cib.3.4.11887 20798827 PMC2928319

[B26] Heldstab S. A. Isler K. Burkart J. M. van Schaik C. P. (2019). Allomaternal care, brains and fertility in mammals: who cares matters. Behav. Ecol. Sociobiol. 73:71. 10.1007/s00265-019-2684-x

[B27] Herbert-Read J. E. (2016). Understanding how animal groups achieve coordinated movement. J. Exp. Biol. 219, 2971–2983. 10.1242/jeb.129411 27707862 PMC5091654

[B28] Herbert-Read J. E. Perna A. Mann R. P. Schaerf T. M. Sumpter D. J. Ward A. J. (2011). Inferring the rules of interaction of shoaling fish. Proc. Natl. Acad. Sci. U S A 108, 18726–18731. 10.1073/pnas.1109355108 22065759 PMC3219133

[B29] Isler K. van Schaik C. P. (2009). Why are there so few smart mammals (but so many smart birds)? Biol. Lett. 5, 125–129. 10.1098/rsbl.2008.0469 18842563 PMC2657741

[B30] Isler K. van Schaik C. P. (2012). Allomaternal care, life history and brain size evolution in mammals. J. Hum. Evol. 63, 52–63. 10.1016/j.jhevol.2012.03.009 22578648

[B31] Jablonski D. (2004). Extinction: past and present. Nature 427:589. 10.1038/427589a 14961099

[B32] Jekabsons M. B. Nicholls D. G. (2004). in situ respiration and bioenergetic status of mitochondria in primary cerebellar granule neuronal cultures exposed continuously to glutamate. J. Biol. Chem. 279, 32989–33000. 10.1074/jbc.M401540200 15166243

[B33] Jungenitz T. Beining M. Radic T. Deller T. Cuntz H. Jedlicka P.. (2018). Structural homo- and heterosynaptic plasticity in mature and adult newborn rat hippocampal granule cells. Proc. Natl. Acad. Sci. U S A 115, E4670–E4679. 10.1073/pnas.1801889115 29712871 PMC5960324

[B34] Keijzer F. (2015). Moving and sensing without input and output: early nervous systems and the origins of the animal sensorimotor organization. Biol. Philos. 30, 311–331. 10.1007/s10539-015-9483-1 26005236 PMC4438119

[B35] Lange S. C. Winkler U. Andresen L. Byhrø M. Waagepetersen H. S. Hirrlinger J.. (2015). Dynamic changes in cytosolic ATP levels in cultured glutamatergic neurons during NMDA-induced synaptic activity supported by glucose or lactate. Neurochem. Res. 40, 2517–2526. 10.1007/s11064-015-1651-9 26184116

[B36] le Feber J. Stegenga J. Rutten W. L. C. (2010). The effect of slow electrical stimuli to achieve learning in cultured networks of rat cortical neurons. PLoS One 5:e8871. 10.1371/journal.pone.0008871 20111726 PMC2810341

[B37] Madhavan R. Chao Z. C. Potter S. M. (2007). Plasticity of recurring spatiotemporal activity patterns in cortical networks. Phys. Biol. 4, 181–193. 10.1088/1478-3975/4/3/005 17928657 PMC2577584

[B38] Magistretti P. J. Allaman I. (2018). Lactate in the brain: from metabolic end-product to signalling molecule. Nat. Rev. Neurosci. 19, 235–249. 10.1038/nrn.2018.19 29515192

[B39] Maloof A. C. Porter S. M. Moore J. L. Dudas F. O. Bowring S. A. Higgins J. A.. (2010). The earliest Cambrian record of animals and ocean geochemical change. Geol. Soc. Am. Bull. 122, 1731–1774. 10.1130/B30346.1

[B40] Maturana H. (1978). “Cognition,” in Wahrnehmung und Kommunikation, eds Hejl P. M. Köck W. K. Roth G. (Frankfurt: Peter Lang), 29–49.

[B43] Maturana H. R. (1980). “Biology of cognition,” in Autopoiesis and Cognition. Boston Studies in the Philosophy and History of Science, eds H. Maturana and F. J. Varela (Dordrecht: Springer), 1–58. 10.1007/978-94-009-8947-4_5

[B41] Maturana H. (2002). Autopoiesis, structural coupling and cognition: a history of these and other notions in the biology of cognition. Cybern. Hum. Knowing 9, 5–34.

[B42] Maturana H. R. (2008). Anticipation and self-consciousness. Are these functions of the brain? Constructivist Found. 4, 18–20. Available online at: http://constructivist.info/4/1/018.

[B44] Maturana H. R. Varela F. J. (1987). The Tree of Knowledge: The Biological Roots of Human Understanding. Boulder, CO: New Science Library/Shambhala Publications.

[B45] Mayr E. (1961). Cause and effect in biology: kinds of causes, predictability and teleology are viewed by a practicing biologist. Science 134, 1501–1506. 10.1126/science.134.3489.1501 14471768

[B46] McGregor S. Virgo N. (2011). Life and its close relatives. Lect. Notes Comput. Sci. 5778, 230–237. 10.1007/978-3-642-21314-4_29

[B47] Newman M. E. J. (1997). A model of mass extinction. J. Theor. Biol. 189, 235–252. 10.1006/jtbi.1997.0508 9441817

[B48] Novellino A. D’Angelo P. Cozzi L. Chiappalone M. Sanguineti V. Martinoia S. (2007). Connecting neurons to a mobile robot: an *in vitro* bidirectional neural interface. Comput. Intell. Neurosci. 2007:12725. 10.1155/2007/12725 18350128 PMC2266971

[B49] Park C. O. Kupper T. S. (2015). The emerging role of resident memory T cells in protective immunity and inflammatory disease. Nat. Med. 21, 688–697. 10.1038/nm.3883 26121195 PMC4640452

[B50] Pedersen S. F. Kapus A. Hoffmann E. K. (2011). Osmosensory mechanisms in cellular and systemic volume regulation. J. Am. Soc. Nephrol. 22, 1587–1597. 10.1681/ASN.2010121284 21852585

[B51] Rangaraju V. Calloway N. Ryan T. A. (2014). Activity-driven local ATP synthesis is required for synaptic function. Cell 156, 825–835. 10.1016/j.cell.2013.12.042 24529383 PMC3955179

[B52] Robinson M. B. Jackson J. G. (2016). Astroglial glutamate transporters coordinate excitatory signaling and brain energetics. Neurochem. Int. 98, 56–71. 10.1016/j.neuint.2016.03.014 27013346 PMC4969184

[B53] Roux E. (2014). The concept of function in modern physiology. J. Physiol. 592, 2245–2249. 10.1113/jphysiol.2014.272062 24882809 PMC4048084

[B54] Schulz K. Sydekum E. Krueppel R. Engelbrecht C. J. Schlegel F. Schröter A.. (2012). Simultaneous BOLD fMRI and fiber-optic calcium recording in rat neocortex. Nat. Methods 9, 597–602. 10.1038/nmeth.2013 22561989

[B55] Shirakawa T. (2006). Anticipatory behavior and intracellular communication in *Physarum polycephalum*. AIP Conference Proceedings (AIP) 839, 541–546. 10.1063/1.2216665

[B56] Sokoloff L. (2008). The physiological and biochemical bases of functional brain imaging. Cogn. Neurodyn. 2, 1–5. 10.1007/s11571-007-9033-x 19003468 PMC2289249

[B57] Southern J. Pitt-Francis J. Whiteley J. Stokeley D. Kobashi H. Nobes R.. (2008). Multi-scale computational modelling in biology and physiology. Prog. Biophys. Mol. Biol. 96, 60–89. 10.1016/j.pbiomolbio.2007.07.019 17888502 PMC7112301

[B58] Tessadori J. Venuta D. Kumar S. S. Bisio M. Pasquale V. Chiappalone M. (2013). “Embodied neuronal assemblies: a closed-loop environment for coding and decoding studies,” in 2013 6th International IEEE/EMBS Conference on Neural Engineering (NER), (San Diego, CA, USA), 899–902. 10.1109/NER.2013.6696080

[B59] Toloe J. Mollajew R. Kügler S. Mironov S. L. (2014). Metabolic differences in hippocampal “Rett” neurons revealed by ATP imaging. Mol. Cell. Neurosci. 59, 47–56. 10.1016/j.mcn.2013.12.008 24394521

[B60] Trestman M. (2013). The cambrian explosion and the origins of embodied cognition. Biol. Theory 8, 80–92. 10.1007/s13752-013-0102-6

[B61] Ulanowicz R. E. Hannon B. M. (1987). Life and the production of entropy. Proc. R. Soc. London. Ser. B. Biol. Sci. 232, 181–192. 10.1098/rspb.1987.0067

[B62] Uomini N. Fairlie J. Gray R. D. Griesser M. (2020). Extended parenting and the evolution of cognition. Philos. Trans. R. Soc. B Biol. Sci. 375:20190495. 10.1098/rstb.2019.0495 32475334 PMC7293161

[B620] Urien L. Xiao Z. Dale J. Bauer E. P. Chen Z. Wang J. (2018). Rate and temporal coding mechanisms in the anterior cingulate cortex for pain anticipation. Sci. Rep. 8:8298. 10.1038/s41598-018-26518-x 29844413 PMC5974274

[B63] Vergara R. C. Jaramillo-Riveri S. Luarte A. Moënne-Loccoz C. Fuentes R. Couve A.. (2019). The energy homeostasis principle: neuronal energy regulation drives local network dynamics generating behavior. Front. Comput. Neurosci. 13:49. 10.3389/fncom.2019.00049 31396067 PMC6664078

[B64] Villalobos M. E. (2015). Biological roots of cognition and the social origins of mind: autopoietic theory, strict naturalism and cybernetics. University of Edinburgh, United Kingdom. Available online at: http://hdl.handle.net/1842/26004.

[B65] Villalobos M. Ward D. (2015). Living systems: autonomy, autopoiesis and enaction. Philos. Technol. 28, 225–239. 10.1007/s13347-014-0154-y

[B66] Volk A. A. Atkinson J. A. (2013). Infant and child death in the human environment of evolutionary adaptation. Evol. Hum. Behav. 34, 182–192. 10.1016/j.evolhumbehav.2012.11.007

[B67] Weber B. Barros L. F. (2015). The astrocyte: powerhouse and recycling center. Cold Spring Harb. Perspect. Biol. 7:a020396. 10.1101/cshperspect.a020396 25680832 PMC4665076

[B68] Yufik Y. (2013). Understanding, consciousness and thermodynamics of cognition. Chaos Solitons Fractals 55, 44–59. 10.1016/j.chaos.2013.04.010

[B69] Yufik Y. (2019). The understanding capacity and information dynamics in the human brain. Entropy (Basel) 21:308. 10.3390/e21030308 33267023 PMC7514789

[B70] Yufik Y. Friston K. (2016). Life and understanding: the origins of “understanding” in self-organizing nervous systems. Front. Syst. Neurosci. 10:98. 10.3389/fnsys.2016.00098 28018185 PMC5145877

